# Supplementary material for: Therapeutic Effect of Alpha Lipoic Acid in a Rat Preclinical Model of Preeclampsia: Focus on Maternal Signs, Fetal Growth and Placental Function
Source: Antioxidants (Basel). 2024 Jun 16;13(6):730. doi: 10.3390/antiox13060730 (PMC11200649; doi:10.3390/antiox13060730)
Supplement: Supplementary file 1 [file antioxidants-13-00730-s001.zip › Expanded Materials and Methods FV.pdf]

Expanded Materials and Methods

Blood pressure determinations

Maternal systolic blood pressure profiles were recorded throughout pregnancy using a validated noninvasive volume pressure recording device, CODA 2 (Kent Scientific, Torrington, CT, USA) as described previously[1]. To verify the reproducibility of the previously reported model of PE[1], determinations were also made in normotensive Wistar Kyoto (WKY) pregnant females undergoing the same ALA administration protocol (as described under Details of the Experimental Protocol). Measurements were made in conscious rats restrained in a thermal plastic chamber. To minimize distress during the measurements, rats were trained for four weeks to become accustomed to restraint before the study and all readings were carried out by the same operator. In addition to baseline (pre-mating), measurements were made on the following time points: GD1, GD7, GD14 (onset of disease) and GD18 (late gestation, fully-established maternal syndrome). All records were taken in the morning, with five cuff inflations as acclimation followed by 20 measurement cycles. The values were averaged, rare outliers (±2 SD from mean) eliminated, and the SBP for each GD was expressed as the mean of the remaining measurements (from at least 10 remaining values/GD analyzed). The data reported includes all records for each animal throughout the duration of the experiment (not just those determinations made at end point), resulting in a larger n for blood pressure data than for the rest of molecular analyses.

Biochemical analyses of renal function

Prior to sacrifice, urine samples were collected over a 24h period by housing the dams in individual metabolic cages with food and water ad libitum. Maternal body weight and urinary output were recorded, and urine samples were collected and stored at −20°C for subsequent analyses. Blood samples were obtained from the abdominal aorta under anesthesia (thiopental sodium 40 mg/kg body weight, i.p.), followed by euthanasia by intracardiac anesthetic overdose for tissue collection. Aliquots of sera and urine were assayed for creatinine using an enzymatic ultraviolet method (Randox Laboratories, Crumlin, Ireland). Urinary protein content (24h proteinuria) was determined by a standard turbidimetric assay and the creatinine clearance (Cr Cl) was calculated according to the standard formula as described previously[1].

Tissue collection

Tissue samples for histology and molecular analyses were collected from rats euthanized while under anesthesia on GD14 (fetoplacental units) or GD20 (kidney, placenta, fetus and amniotic fluid) as described[1]. Briefly, the uterus was dissected exposing the fetoplacental units, and amniotic fluid was collected by aspiration with a 25G syringe. From each uterine specimen, whole implantation sites (i.e., the placenta with its associated mesometrial triangle and decidual tissue) were carefully separated and processed according to standard methods for histological sectioning, or snap frozen in liquid nitrogen for molecular analyses. Kidneys were dissected, decapsulated and cut longitudinally, followed by fixation in phosphate-buffered 10% formaldehyde (pH 7.2) and paraffin embedding according to our standard protocol.

Renal histology

Morphometric evaluation of renal damage was carried out on 4μm-thick sections of paraffin embedded organs stained by the PAS method. Histological evaluation was performed on 10–15 random fields photographed at ×400 magnification per section, using a Nikon E400 microscope (Nikon Instrument Group, Melville, NY, USA). Stained sections were evaluated by an independent observer for the presence of mesangial matrix expansion, which was estimated as the PAS-positive glomerular area fraction using the thresholder algorithm of the QuPath software (v.0.4.3)[2]. Expression of podocin was evaluated following a standard immunoperoxidase protocol. Briefly, sections were deparaffinized, rehydrated and incubated for 30 min in 3% H_2O_2

in PBS for quenching of endogenous peroxidase activity. The sections were then washed in PBS (pH 7.2) for 20 min, followed by incubation with blocking serum for 20 min. The primary antibody (podocin, sc-21009, Santa Cruz Biotechnology, Dallas, TX, USA; 1:200) was incubated for 1h at room temperature. Sections were then washed and incubated with a biotinylated universal antibody (SS Multilink, Biogenex, Fremont, CA, USA) for 30 min, followed by detection of the signal using a liquid diaminobenzidine (DAB) Substrate Chromogen System (cat. #K3467, DAKO, Darmstadt, Germany). After washing, nuclei were counterstained with 0.1% Mayer's hematoxylin, followed by a standard dehydration procedure and mounting in a DPX histology medium (Millipore-Sigma, St. Louis, MO, USA). Photo documentation was performed as described above and podocin expression was determined as the DAB-positive glomerular area fraction using the QuPath thresholder.

<u>Evaluation of fetal development</u>

Morphometric analysis of fetal development was performed on GD20. Briefly, following collection of amniotic fluid, the amnion was dissected to expose the fetuses and placentas, which were fixed in phosphate-buffered 10% formaldehyde (pH 7.2). Placental and fetal weights were recorded from the fixed specimens and fetal head diameter measured using a Vernier caliper. The cephalization index (head circumference to body weight ratio) was calculated as an indicator of asymmetric fetal growth as described in our previous publication[1].

<u>Placental histology and immunohistochemistry</u>

Paraffin embedded implantation sites were sectioned (4-μm thick) parallel to the mesometrial-fetal axis, and stained with PAS. For immunolabeling, we followed our standard protocol as described above for kidney sections using a polyclonal anti ACTA2 (1:200, MU128-UC, Biogenex, Fremont, CA, USA) incubated for 30 min at room temperature as primary antibody. Isolectin B4 (IB4) binding was assessed as described previously using biotin-conjugate Isolectin GS-IB4 From Griffonia simplicifolia (I21414, Invitrogen, Waltham, MA, USA)[1].

Spiral artery remodeling was evaluated in sets of ten serial parallel sections stained with ACTA2. Briefly, images were acquired at x400 magnification using the Nikon E400 microscope (Nikon Instrument Group, Melville, NY, USA) so that all arterial cross sections in the mesometrial triangle of each placental sample were documented. Then, the arterial wall area was delimited for each cross-section based on hematoxylin staining using the brush selection tool in QuPath, followed by running of the pixel thresholder algorithm to determine the arterial wall ACTA2 content as the DAB-positive area fraction. For quantification of glycogen trophoblasts in PAS-stained sections, 10-15 random fields within the placental junctional zone were imaged at x200 magnification. The images were evaluated by an independent observer for determination of the PAS-positive area fraction using the thresholder algorithm of QuPath software. Analysis of vascularization of the labyrinth was performed on IB4 stained sections using Angiotool as described previously[1,3].

<u>Western blots</u>

Placental protein expression was determined following our previously published western blot protocol[4]. Briefly, samples were homogenized in lysis buffer (150 mM NaCl, 50 mM Trizma–HCl, 1% NP-40, pH 8.0) in the presence of protease and phosphatase inhibitors and centrifuged at 600x g for 20 min. The samples containing the same protein concentration were added with a 2X solution of the sample buffer (62.5 mM Tris–HCl, pH 6.8 2% SDS, 25% glycerol, 5% β-mercaptoethanol and 0.01% bromophenol blue) and heated at 95 °C for 2 min. Sample aliquots (30 μg protein) were separated by reducing 10% polyacrylamide gel electrophoresis and electro transferred to PVDF membranes. Colored molecular weight standards (GE Healthcare, Piscataway, NJ, USA) were run simultaneously. Primary antibodies were purchased to Santa

Cruz Biotechnology (Dallas, TX, USA) (gp91phox #sc130543, p47phox #sc17844, SOD2 #sc133134, ACTB #sc47778, GADPH #sc47724), to Abcam (Cambridge, UK) (NOX1 #ab131088, and NOXO1 #ab34761), and Invitrogen (Waltham, MA, USA) (NLRP3 # PA5-79740).

Membranes were blotted for 2 h in 5% (w/v) non-fat milk at room temperature, and incubated overnight in the presence of the primary antibodies (1:1000 dilution) in 5% (w/v) bovine serum albumin in TBS buffer (50 mM Tris, 150 mM NaCl, pH 7.6), containing 0.1% (v/v) Tween-20 at 4°C. After incubation for 1 h in the dark at room temperature in the presence of the biotin-conjugated secondary antibody (anti-rabbit IgG H&L #ab97049 or anti-mouse IgG H&L #ab97021, Abcam, Cambridge, UK) (1:15000 dilution) in TBS buffer, membranes were incubated with streptavidin peroxidase polymer ultrasensitive (#S2438, Sigma Aldrich, St. Louis, MO, USA) (1:15000) in TBS buffer for 1 h in the dark at room temperature. Peroxidase activity was detected using a chemiluminescence kit (BioRad, Hercules, CA, USA). The films were scanned and a densitometric nalysis was performed using ImageJ (National Insitutes of Health, Bethesda, MD, USA). Band densities were normalized to the GADPH or ACTB content.

Placental gene expression analysis

Total RNA was extracted from placental tissue using Trizol (Invitrogen, Waltham, MA, USA). Quantification of placental mRNA expression was performed using a real-time reverse transcriptase polymerase chain reaction protocol normalized by beta actin (*Actb*) housekeeping gene expression as described previously[5].For cDNA synthesis,2 µg of total RNA and 1 µg of random hexamer primers (Promega, Madison, WI, USA) were heated followed by incubation at 4˚C. The reverse transcription reaction was run by adding 5 µl of MMLV RT 5X buffer (250 mmol/l Tris-HCl, pH 8.3, 375 mmol/l KCl, 15 mmol/l MgCl2,50 mmol/l DTT), 25 U RNasin (Promega Corp, Madison, WI, USA), 1.25 µl of 10 mmol/l dNTPs (Invitrogen, Waltham, MA, USA), and 200 U of MMLV-reverse transcriptase (Promega Corp, Madison, WI, USA) and incubating at 37 °C for 1 h, followed by inactivation of the enzyme at 95 °C for 10 min. Then, real-time PCR was performed using a Bio-Rad iQ iCycler Detection System (BioRad Laboratories, Hercules, CA, USA) with SYBR green fluorophore. Reactions (in duplicate) were carried out in a total volume of 20 µl. A 3-step protocol (95˚C for 30 seconds; 65˚ C annealing for 30 seconds; and 72˚C extension for 40 seconds) was run for 46 cycles. Primers (Invitrogen, Waltham, MA, USA) for rat caspase 1 (*Casp1*), interleukin 1 beta (*Il1b*), interleukin 18 (*Il18*), Flt1 (*Flt1*), vascular endothelial growth factor (*Vegfa*), hypoxia inducible factor alpha (*Hif1a*) and actin beta (*Actb*) were designed as shown in Table 1.

Table S1. Primer sequences

Gene	Forward	Reverse	Product length (bp)
Casp1 (NM_012762.3)	5′-GGAGCTTCAGTCAGGTCCAT-3′	5′-CGCCACCTTCTTTGTTCAGT-3′	110
Il1b (NM_031512.2)	5′-AGCAGCTTTCGACAGTGAGG-3′	5′-TGCCTTCCTGAAGCTCTTGT-3′	157
Il18 (NM_019165.2)	5′-AATCACTTCCTCTTGGCCCA-3′	5′-CCAAGTCTCCATTATCTTCAGGT-3′	126
Flt1 (NM_019306.2)	5′-CGGCAGACCAATACAATCCT-3′	5′-TGTATTGAGGTCCGTGGTGA-3′	111

Vegfa (NM_031836.3)	5′-GCCCATGAAGTGGTGAAGTT-3′	5′-ACTCCAGGGCTTCATCATTG-3′	172
Hif1a (NM_024359.2)	5′-CGATGACACGGAAACTGAAG-3′	5′-TTCGAAGTGGCTTTGGAGTT-3′	143
Actb (NM_031144.3)	5′-TTCCTGGGTATGGAATCCTG-3′	5′-CAGCAATGCCTGGGTACAT-3′	136

A melt curve analysis was performed after every run to ensure a single amplified product for every reaction. The size of the amplicons generated was confirmed on a 2% agarose gel. We did not find any significant differences between experimental groups in the *Actb* housekeeping gene by the GeNorm software. Quantification was performed by normalizing Ct (cycle threshold) values with *Actb* Ct and analyzed with the $2^{-\Delta\Delta CT}$ method.

Determination of Thiobarbituric Acid Reactive Substances (TBARS)

The assay was performed following a previously described method[4]. Briefly, the samples were mixed with 3% sodium dodecyl sulfate (SDS), 0.1 N HCl, 10% phosphotungstic acid and 0.7%2-thiobarbituric acid and heated for 45 min in boiling water; TBARS were extracted with 2 mL of n-butanol and detected fluorometrically (λ_{ex}: 515 nm; λ_{em}: 555 nm). To prepare the standard of malondialdehyde, 1,1,3,3-tetramethoxypropane was used. The results were expressed as TBARS nmol (malondialdehyde equivalents)/mg protein

ELISA

Commercial ELISA kits were used for determination of IL18 (#KRC2341, Invitrogen, Waltham, MA, USA) in amniotic fluid samples and sFlt1 (#MVR100, R&D Systems, Inc., MN, USA) levels in maternal serum. Assays were run following the manufacturer instructions.

Statistical Analysis

Results are expressed as means±SD or median (minimum-maximum), with n representing the number of individual experiments, pregnant dams, or fetuses. The normal distribution of samples was assessed using the Shapiro-Wilk normality test. Student's T-test or Mann-Whitney U-tests were used to analyze normally distributed and skewed data respectively. Data sets in which comparisons included WKY pregnancies as controls were analyzed by two-way ANOVA with factors group (WKY vs. SHRSP) and ALA treatment, followed by Bonferroni post-hoc tests. Graphing and calculations were performed using the GraphPad Prism 8 software. A p-value <0.05 was considered statistically significant

Details of the Experimental Protocol

Experimental design

We conducted an exploratory study with the aim to evaluate the therapeutic potential of ALA as an intervention in PE by assessing the effect of treatment on maternal signs, fetal growth and placental function in pregnant Stroke-Prone Spontaneously Hypertensive Rats (SHRSP). The study compared two groups of SHRSP animals: ALA-treatment, receiving 25mg/kg ALA (Laboratorios Gador, Buenos Aires, Argentina) injected i.p. on GD1, GD8 and GD12 and controls (receiving saline following the same protocol). The timing of administration was set empirically

to encompass the placentation period (which is completed by GD12) while minimizing animal distress due to manipulation during the peri-implantation period. The dose used was set in agreement with previous reports on the administration of ALA to pregnant rats [6]. To verify reproducibility of the PE-like syndrome described in our previous publications using the SHRSP model[1], blood pressure profiles and fetal growth parameters were also recorded in normotensive WKY dams undergoing the same ALA administration protocol.

Inclusion and Exclusion criteria

Nulliparous SHRSP females of 10-12 weeks of age, weighing 200-250 g were allocated to the study upon detection of the vaginal plug (GD1). Animals that were not pregnant were excluded at the time of surgery for tissue collection. Breeding efficiency (%plug-positive females that were pregnant) was 89% for the ALA-treatment group and 84% for the control treatment.

Randomization

Females were randomly paired to littermate males (at a 1:1 ratio) using a computer based random order generator to establish timed pregnancies. Upon plug detection, allocation to the treatment groups was performed using a minimization strategy so that there was a balanced distribution of animals to the treatment groups throughout the duration of the study.

Blinding

Plug detection and blood pressure measurements were performed by a technician different from the one that administered the treatments and assisted on the surgery. The treatment was prepared by a third operator who blinded the content of the tubes with a number.

Sample size and power calculation

The number of animals included was based on previously published studies [1,4] using the same model and met the logistic limitations of the animal facility.

Animal source

The animals were SHRSP, 10–12-weeks-old, weighing between 200g and 250g before mating. They were obtained from the breeding colony maintained in the animal facility of Laboratorio de Medicina Experimental, which was established from animals purchased from Charles River Laboratories (Wilmington, MA, USA).

Number of animals per group

Matings were established so as to achieve an n of 8-10 animals per treatment group for each time point (GD14 and GD20). At the time of surgery, 8 animals from the SHRSP control group and 5 from the ALA treatment group were not pregnant and were excluded from the study. At the end of the experiment, a total number of 39 WKY and 35 SHRSP females were used, as follows:

Table S2. Number of animals per group

Group	GD14 (n)	GD20 (n)	Total pregnant
WKY no treatment	10	10	20
WKY ALA	10	9	19
SHRSP no treatment	10	9	19
SHRSP ALA	8	8	16

Main outcome measurements

1. Raw data systolic blood pressure (in mmHg)

Systolic blood pressure profiles were recorded by one experienced technician using the CODA 2 (Kent Scientific, Torrington, CT, USA) tail-cuff system. Measurements were taken on the morning, at baseline (pre-mating) and on GD1, GD7, GD14 and GD18, by averaging 15-20 individual measures for each animal, as shown in Table 1. Measurements were made in conscious rats restrained in a thermal plastic chamber, in which the animal was allowed to sit for 10 minutes before the recording. To minimize distress during the procedure, rats were trained for four weeks to become accustomed to restraint before the study.

Table S3. Blood pressure data

	WKY			WKY ALA			SHRSP			SHRSP ALA		
	Mean	SD	N	Mean	SD	N	Mean	SD	N	Mean	SD	N
Baseline	115.7	15.5	20	112.5	14.5	19	154.7	10.9	19	148.6	13.5	16
GD7	120.2	14.1	20	118.4	13.7	17	166.0	15.3	19	151.4	15.7	15
GD14	115.1	15.7	19	111.0	14.1	19	169.3	19.4	18	147.2	13.4	16
GD18	108.7	13.0	10	117.1	9.8	7	182.8	10.1	9	142.9	9.3	7

2. Raw data fetal weight (in g)

Morphometric analysis of fetal development was performed on GD20. The uterine horns were cut longitudinally to expose the fetoplacental units. After collection of amniotic fluid, the amnion was dissected to expose the fetuses and placentas, which were fixed in phosphate-buffered 10% formaldehyde (pH 7.2). Placental and fetal weights were recorded from the fixed specimens. Data were expressed as the litter mean for each dam (Table 2).

Table S4: Fetal weights

	SHRSP no treatment			SHRSP ALA		
Litter	Mean	SD	N	Mean	SD	N
1	1.72	0.13	13	1.95	0.06	14
2	1.84	0.14	12	1.97	0.19	8
3	1.61	0.05	8	1.97	0.09	13
4	1.82	0.14	11	1.93	0.17	13
5	2.02	0.11	12	1.99	0.07	11
6				2.04	0.16	7
7				2.33	0.31	11

<u>Experimental details, ethics, and funding statements</u>

The experiments were performed between March 2021 and April 2022 at the animal facility of the Laboratorio de Medicina Experimental (Hospital Alemán). Ethical approval was obtained from the Institutional Animal Care Committee (CICUAL, Facultad de Medicina, Universidad de Buenos Aires, License 2064/2015 to GB). The study was partially funded by the Agencia Nacional de Promoción de la Investigación, el Desarrollo Tecnológico y la Innovación (Agencia I+D+I, grant number: PICT-2020-03263 to GB), and therefore satisfies its criteria regarding the use of live animals for scientific research purposes.

References

1. Barrientos, G.; Pussetto, M.; Rose, M.; Staff, A.C.; Blois, S.M.; Toblli, J.E. Defective trophoblast invasion underlies fetal growth restriction and preeclampsia-like symptoms

in the stroke-prone spontaneously hypertensive rat. *Molecular human reproduction* **2017**, *23*, 509-519, doi:10.1093/molehr/gax024.
2. Bankhead, P.; Loughrey, M.B.; Fernandez, J.A.; Dombrowski, Y.; McArt, D.G.; Dunne, P.D.; McQuaid, S.; Gray, R.T.; Murray, L.J.; Coleman, H.G.; et al. QuPath: Open source software for digital pathology image analysis. *Sci Rep* **2017**, *7*, 16878, doi:10.1038/s41598-017-17204-5.
3. Zudaire, E.; Gambardella, L.; Kurcz, C.; Vermeren, S. A computational tool for quantitative analysis of vascular networks. *PloS one* **2011**, *6*, e27385, doi:10.1371/journal.pone.0027385.
4. Blois, S.M.; Prince, P.D.; Borowski, S.; Galleano, M.; Barrientos, G. Placental Glycoredox Dysregulation Associated with Disease Progression in an Animal Model of Superimposed Preeclampsia. *Cells* **2021**, *10*, doi:10.3390/cells10040800.
5. Schuman, M.L.; Peres Diaz, L.S.; Aisicovich, M.; Ingallina, F.; Toblli, J.E.; Landa, M.S.; Garcia, S.I. Cardiac Thyrotropin-releasing Hormone Inhibition Improves Ventricular Function and Reduces Hypertrophy and Fibrosis After Myocardial Infarction in Rats. *J Card Fail* **2021**, *27*, 796-807, doi:10.1016/j.cardfail.2021.04.003.
6. Ghoneim, F.M.; Alrefai, H.; Elsamanoudy, A.Z.; Abo El-Khair, S.M.; Khalaf, H.A. The Protective Role of Prenatal Alpha Lipoic Acid Supplementation against Pancreatic Oxidative Damage in Offspring of Valproic Acid-Treated Rats: Histological and Molecular Study. *Biology (Basel)* **2020**, *9*, doi:10.3390/biology9090239.
