# Supplementary material for: Therapeutic Effect of Alpha Lipoic Acid in a Rat Preclinical Model of Preeclampsia: Focus on Maternal Signs, Fetal Growth and Placental Function
Source: Antioxidants (Basel). 2024 Jun 16;13(6):730. doi: 10.3390/antiox13060730 (PMC11200649; doi:10.3390/antiox13060730)
Supplement: Supplementary file 1 [file antioxidants-13-00730-s001.zip › Supplemental Figures FV.pdf]

Supplemental Figures

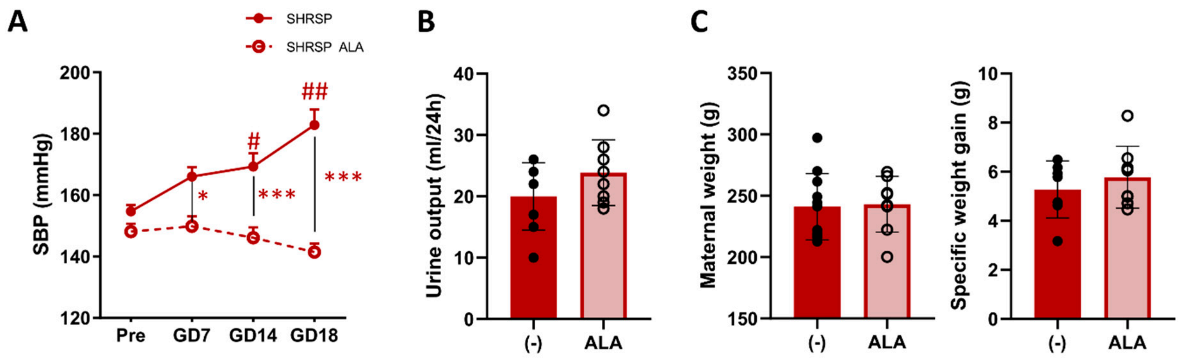

Figure S1. Effect of ALA supplementation on maternal signs (A) Effect of ALA supplementation on the systolic blood pressure (SBP) profile recorded throughout pregnancy. Data are expressed as mean±SEM, n=19-16/group. Statistical significance denoted as ##p<0.01, #p<0.05 vs. baseline (pre) and ***p<0.001, *p<0.05 control vs. ALA-treated SHRSP analyzed by 2-way ANOVA and Tukey post-hoc test. (B) Maternal urine output, assessed on GD20. Data are individual measurements and mean±SD, n=6-9/group. (C) Maternal weight (left graph) and specific weight gain (calculated as ($Weight_{GD20}$-$Weight_{GD1}$)/litter size, right graph) determined on GD20. Data are individual measurements and mean±SD, n=8 animals/group.

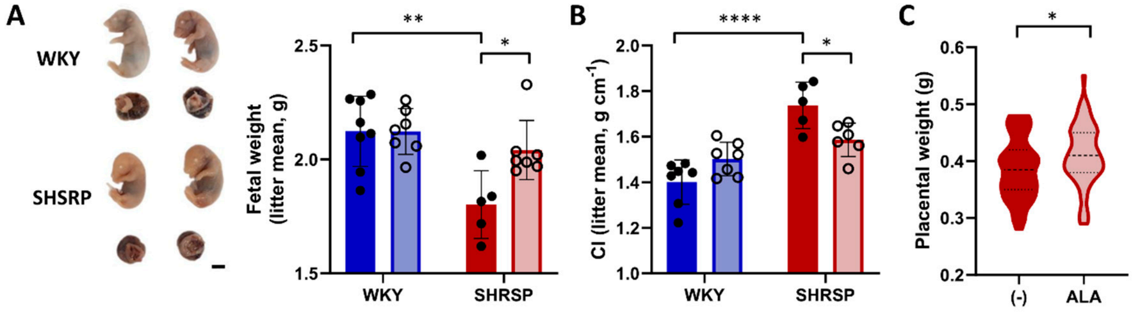

Figure S2. Effect of ALA supplementation on fetal phenotype. (a) Representative examples of formalin-fixed fetal and placental specimens collected on GD20 from WKY and SHRSP dams. Scale bar: 5 mm. Treatment with ALA restored fetal growth in SHRSP pregnancies, as noted by the increased fetal weights in ALA treated dams (bar graph), which showed no differences compared to WKY. Data are individual measurements and mean±SD, n=5-10 litters/group. **p<0.01, *p<0.05 analyzed by 2-way ANOVA and Tukey post-hoc test. (b) Effect of ALA supplementation on asymmetric FGR, as estimated with the Cephalization index (CI). Data are individual measurements and mean±SD, n=5-10 litters/group. ****p<0.0001, *p<0.05 analyzed by 2-way ANOVA and Tukey post-hoc test. (c) Placental weights measured in ALA-supplemented SHRSP on GD20. Data are medians (dashed line) and minimum-maximum, n=53-54 specimens/group. *p<0.05 analyzed by T test.

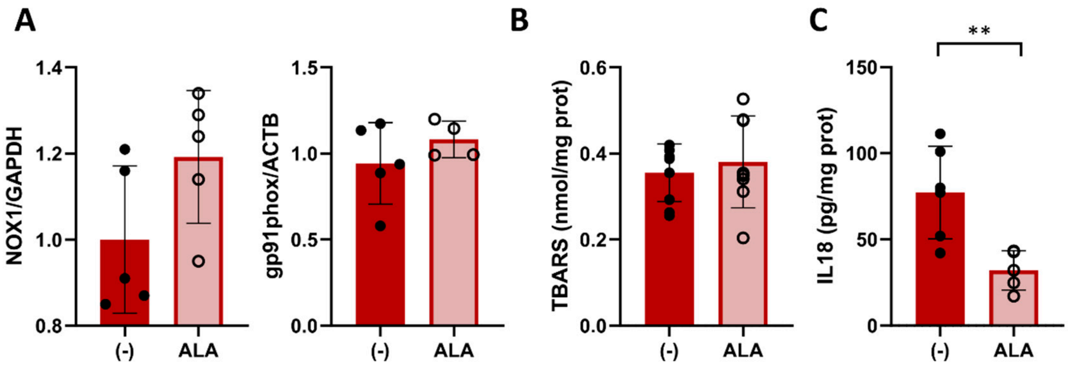

Figure S3. **Analysis of oxidative stress and inflammation markers in placenta and amniotic fluid** (A) Western blot analysis of placental expression of NOX subunits NOX1 (left graph) and gp91phox (right graph) on GD14. Data are individual values and mean±SD, n=4-5 placentas/group, analyzed by Mann-Whitney U-test. (B) Levels of thiobarbituric acid reactive substances (TBARS) measured in amniotic fluid samples collected on GD20. Results expressed as individual measurements and mean±SD, n=9-8 samples/group (C) Amniotic fluid levels of IL18, determined by ELISA. Treatment with ALA decreased intraamniotic IL18 concentrations in SHRSP pregnancies. Data are individual measurements and mean±SEM, n=5-7 samples/group, **p<0.01 analyzed by T test.
