# Supplementary figures and images for: Therapeutic Effect of Alpha Lipoic Acid in a Rat Preclinical Model of Preeclampsia: Focus on Maternal Signs, Fetal Growth and Placental Function
Source: Antioxidants (Basel). 2024 Jun 16;13(6):730. doi: 10.3390/antiox13060730 (PMC11200649; doi:10.3390/antiox13060730)

# Full unedited blots

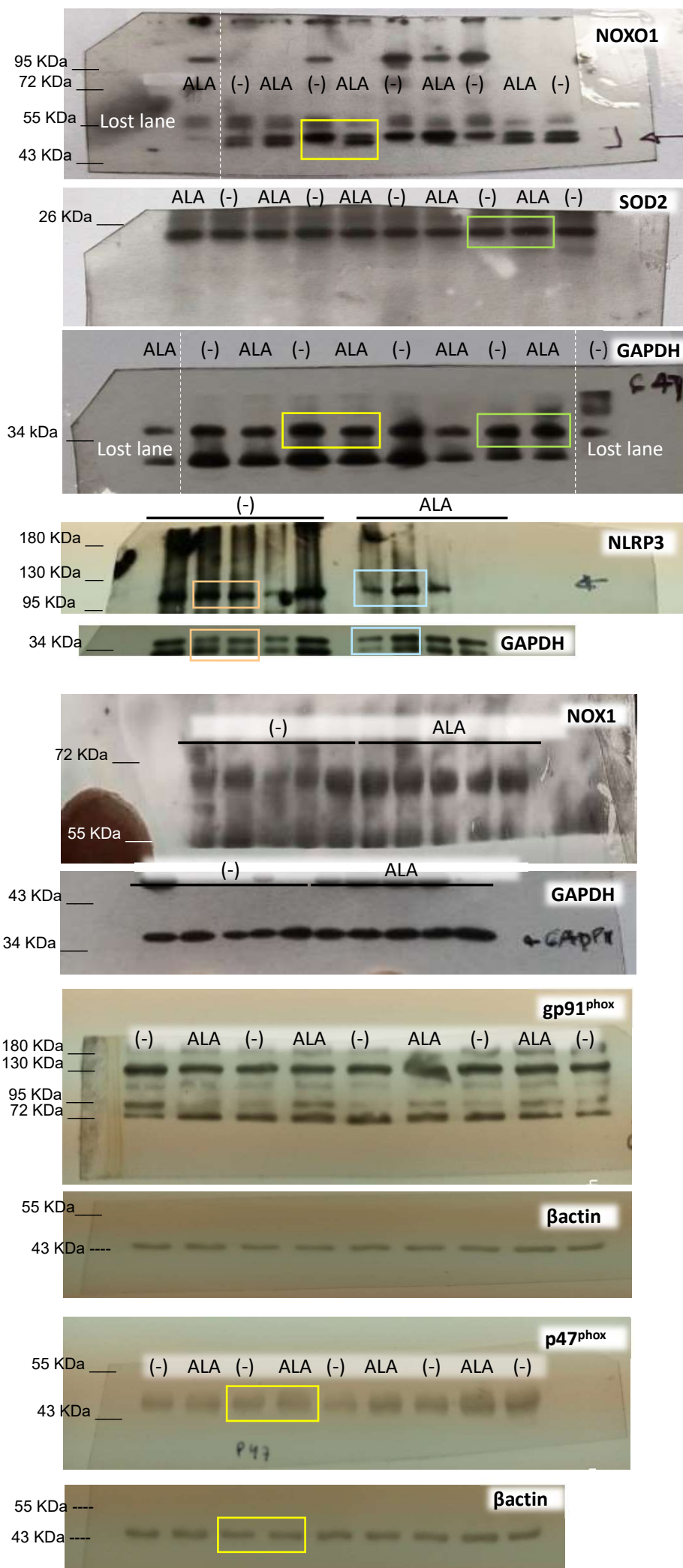

Supplement: Supplementary file 1 [file antioxidants-13-00730-s001.zip › Full unedited blots for Fig 3.pdf]
